# Pyrazole-Imidazoline Derivative Prevents Cardiac Damage and Mortality in Acute *Trypanosoma cruzi* Infection

**DOI:** 10.3390/ph18101552

**Published:** 2025-10-15

**Authors:** Lorraine Martins Rocha Orlando, Leonardo da Silva Lara, Thamyris Pérez de Souza, Vitoria Barbosa Paes, Claudia Magalhães Calvet, Liliane Batista de Mesquita, Guilherme Cury Lechuga, Cynthia Nathália Pereira, Maurício Silva dos Santos, Mirian Claudia de Souza Pereira

**Affiliations:** 1 Laboratório de Ultraestrutura Celular, Instituto Oswaldo Cruz, Fiocruz. Av. Brasil 4365, Rio de Janeiro 21040-900, RJ, Brazil; lorrainemartins07@hotmail.com (L.M.R.O.); leonardosilva.lara@hotmail.com (L.d.S.L.); perezthamyris@gmail.com (T.P.d.S.); vitpaes@gmail.com (V.B.P.); cmcalvet@ioc.fiocruz.br (C.M.C.); lilianemesquita@ioc.fiocruz.br (L.B.d.M.); gclechuga@gmail.com (G.C.L.); 2 Laboratório de Síntese de Sistemas Heterocíclicos (LaSSH), Instituto de Física e Química (IFQ), Universidade Federal de Itajubá, Av. BPS 1303, Pinheirinho, Itajubá 37500-903, MG, Brazil; cynthianathpereira@yahoo.com.br (C.N.P.); mauriciosantos@unifei.edu.br (M.S.d.S.)

**Keywords:** *Trypanosoma cruzi*, Chagas disease, pyrazole, fibrosis

## Abstract

**Background:** Chagas disease poses a significant public health challenge, particularly impacting socioeconomically vulnerable populations. Current treatment strategies still rely on two nitro heterocyclic compounds: benznidazole and nifurtimox. Both agents exhibit limited therapeutic efficacy during the chronic phase of the disease and are often linked to severe adverse effects that frequently lead to treatment discontinuation. This urgent need for safer, more effective oral treatments drives the development of novel chemotypes. **Objective:** In this study, we advanced the preclinical evaluation of 4-imidazoline-1*H*-pyrazole derivatives, which have been identified as promising candidates against *Trypanosoma cruzi*. **Methods:** The candidate compound identified from the reversibility assay underwent further evaluation for its efficacy using a three-dimensional (3D) culture model and a Transwell co-culture system, in addition to the in vivo assessment. **Results:** Our findings revealed that compound **3m** (3-Cl, 4-CH_3_) exhibited low cytotoxicity while substantially decreasing the parasite burden in 3Dcardiac spheroid models. The compound effectively permeated Caco-2 cell monolayers and demonstrated the ability to inhibit *T. cruzi* infection in Vero cell cultures within a co-culture system. Furthermore, the **3m** derivative not only controlled parasite resurgence but also showed significant therapeutic benefits in a murine model of acute *T. cruzi* infection, resulting in marked reductions in parasitemia and tissue parasitism, associated with diminished inflammatory infiltrate and cardiac fibrosis. Treatment with **3m** increased the survival rate of infected mice to 40%, comparable to the reference drug benznidazole in several key pathological endpoints. **Conclusion:** These findings highlight the potential of 4-imidazoline-1*H*-pyrazole derivatives, particularly compound **3m**, in mitigating the pathological effects associated with *T. cruzi* infection.

## 1. Introduction

Chagas disease (CD), caused by the protozoan *Trypanosoma cruzi*, poses a significant global public health challenge. Recognized as a neglected tropical disease by the World Health Organization (WHO), CD’s impact extends beyond the borders of endemic countries in Latin America due to the migration of infected individuals to non-endemic countries, where more than 4 million migrants reside in Europe [1]. Approximately 7 million people worldwide are infected, with 75 million at risk of contracting the disease [2]. Despite successful control of vector and blood transfusion routes in many endemic countries, the disease reemerges in outbreaks of oral infection, presenting increased clinical severity and mortality rates [3,4]. Mother-to-child transmission also facilitates the silent spread of CD, with cases of congenital CD reported in non-endemic countries, particularly in Spain [5]. This often-asymptomatic disease in its acute phase may result in cardiovascular or digestive damage in 30–40% of CD carriers. Chronic chagasic cardiomyopathy, characterized by low-grade parasitism, inflammation, and fibrosis, is the leading cause of heart failure in patients from Latin America, with an annual mortality rate of 8% [6].

Antiparasitic therapies rely on benznidazole (Bz) and nifurtimox (Nif), both nitro heterocyclic drugs discovered over five decades ago. The efficacy of these medications ranges from 60% to 80% during the acute phase but drops to 20% to 40% cure rates in the chronic phase [7,8]. The treatment regimens for both drugs are lengthy, typically lasting 60 to 90 days, and they often lead to severe adverse effects, resulting in treatment discontinuation (5% to 31%) [7]. Low treatment coverage, currently less than 1%, poses a significant barrier that needs to be overcome. Developing new, effective, and safe drugs remains a key challenge. Despite the advancement of potential candidates into clinical trials over the last few decades, there has been a lack of successful outcomes, often marked by therapeutic failure or low tolerability [9,10,11]. Posaconazole, a potent antifungal agent that inhibits ergosterol biosynthesis, did not exhibit sustained effectiveness in monotherapy [9] and showed no advantage in benznidazole-combined therapy [12]. The fexinidazole clinical trial was halted due to neutropenia and hepatotoxicity [11]. Alternative treatment regimens involving low doses and short durations of benznidazole have shown the most promising results to date, with the potential to enhance patient adherence to treatment and reduce treatment abandonment [13]. Despite the confirmed efficacy of the alternative low-dose, short-duration Bz regimen in the MULTIBENZ trial, concerns persist regarding the Brazilian population, which has shown a poor response to both standard and alternative Bz treatment regimens [14]. Various compound classes have shown potential for antiparasitic activity, but only a few novel candidates have progressed through the preclinical and clinical pipelines for CD [15,16,17].

In this scenario, we focused on optimizing a 5-amino-4-imidazoline-1*H*- pyrazole hit identified as a cruzain inhibitor [18]. Pyrazole, a heterocyclic compound containing a five-membered ring with two nitrogen atoms, has demonstrated promise as an antiparasitic agent [19,20]. This scaffold selection was based on its known efficacy as a potent inhibitor of *T. cruzi* cruzain, a well-characterized therapeutic target for Chagas disease [18]. Our research identified two pyrazole-imidazoline derivatives that demonstrated activity against both the trypomastigote and intracellular amastigote stages of *T. cruzi* [18]. Regarding their mechanism of action, the most effective analogs, specifically 3,4- and 3,5-dichloro derivatives of the pyrazole-imidazoline series, exhibited binding affinity for the active site of *T. cruzi* cruzain. These compounds were effective in inhibiting the parasite’s protease activity by over 50%, emphasizing their potential as a promising therapeutic candidate [18]. The optimization strategy explored the structure-activity relationship by introducing electron-withdrawing and electron-donating groups. This included adding functional groups, such as carboxamide and methyl groups present in the compound N-(1*H*-benzimidazole-2-yl)-1,3-dimethyl-1*H*-pyrazole-4-carboxamide (3H5), described in the Protein Data Bank (PDB) as an inhibitor of cruzain [21]. Of the five new pyrazole series examined, the 4-imidazoline-1*H*-pyrazole derivatives (series 3) exhibited higher activity against intracellular amastigotes compared to the pyrazole-carboxamide series (series 1 and 2) and the 5-amino-4-imidazoline-1*H*-pyrazole derivatives with a methyl group in the pyrazole (series 4) or imidazoline (series 5) ring [22]. The three most active candidates selected, **3g** (4-Cl), **3j** (4-Br), and **3m** (3-Cl, 4-CH_3_), exhibited high in vitro pIC50 (negative log of IC_50_ in molar) values, ranging from 5.22 to 5.56, against intracellular amastigotes and showed greater potential in controlling parasite load in 3D spheroids of Vero cells and short-term reversibility assays. In this study, we expanded the analysis of these 4-imidazoline-1*H*-pyrazole derivatives by implementing new approaches to our screening platform, thereby advancing the efficacy analysis in an acute *T. cruzi* infection model. Our findings indicate that treatment with **3m** significantly improved survival rates in animal models by reducing tissue parasitism, inflammation, and cardiac fibrosis.

## 2. Results and Discussion

### 2.1. Effect of 3m Treatment on In Vitro Parasite Resurgence

**Figure 1:**
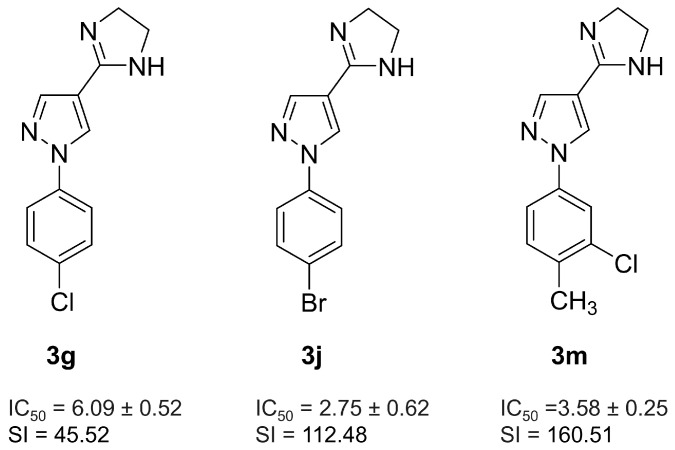
Chemical structures of **3g**, **3j**, and **3m**. The 50% inhibitory concentration (IC_50_) values and selectivity index (SI) represent activity against *T. cruzi* intracellular amastigotes.

Our previous investigation identified three 4-imidazoline-1*H*-pyrazole derivatives, **3g**, **3j**, and **3m** (Figure 1), that exhibit potential antiparasitic activity [22]. These promising initial findings have led to more detailed in vitro preclinical assays to evaluate their therapeutic efficacy and safety profiles. To reduce the risk of late-stage drug failure, improvements to the drug discovery screening cascade have been recommended [23,24]. Therefore, we expanded our research by incorporating advanced cell-based in vitro models to assess these compounds further.

**Figure 2:**
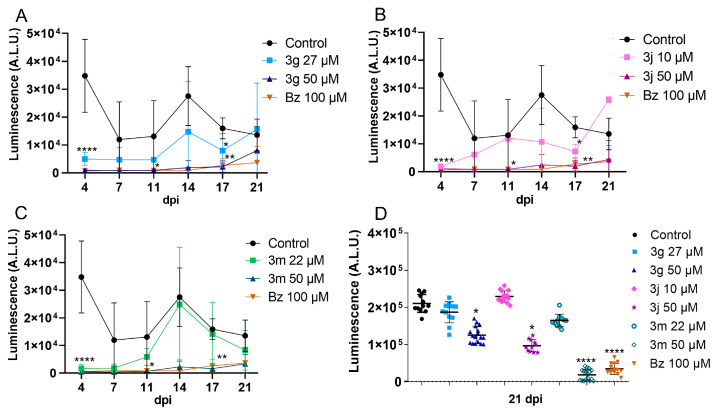
Effect of **3g**, **3j,** and **3m** on the resurgence of the parasite in vitro. Vero cells infected with *T. cruzi*, Dm-28c-Luc, were treated with pyrazole-imidazoline derivatives (maximum concentration of 50 µM) or Bz (100 µM) for 10 days, followed by an additional 10 days without treatment. (**A**–**C**) The analysis of trypomastigote release in the culture supernatant was conducted after treatment with **3g** (**A**), **3j** (**B**), and **3m** (**C**). Additionally, parasite viability in Vero cell monolayers at 21 days post-infection (dpi) was monitored by adding the luciferase enzyme substrate (luciferin at 300 µg/mL; **D**). DMSO and Bz served as controls. At the highest concentration (50 µM), **3j** and **3m** inhibited the release of parasites to levels comparable to Bz. In the cell monolayer, **3j** (50 µM) and **3m** (both at 22 µM and 50 µM) effectively reduced the parasite load. Data are presented as mean ± standard deviation from three independent experiments, each with at least four replicates. Statistical significance was determined by One-way ANOVA followed by Tukey’s post hoc test, with significance levels indicated at *p* ≤ 0.05 (*), *p* ≤ 0.01 (**) and *p* ≤ 0.0001 (****).

Since these pyrazole-imidazoline derivatives demonstrated sustained inhibition of infection reactivation in a short-term washout assay [22], our initial strategy focused on evaluating these compounds over longer treatment periods. A 10-day treatment was conducted to evaluate the resurgence of parasitism after withdrawing these compounds. The three derivatives assessed (**3g**, **3j**, and **3m**) exhibited different effects on trypomastigote release, with 50 µM identified as the optimal concentration. At the 90% inhibitory concentrations (IC_90_), the derivatives did not suppress parasite release, resulting in increased release after the compounds were removed (Figure 2). In contrast, treatment with 50 µM of the pyrazole-imidazoline derivatives (**3g**, **3j**, and **3m**) yielded results comparable to those of benznidazole (Bz). Derivatives **3j** and **3m**, and also Bz, exhibited a 92% and 94% reduction in parasite release over a 21-day observation period (Figure 2). The infection profile of the cell monolayer was assessed at the end of the washout assay (21 days). Derivatives **3g**, **3j**, and **3m**, administered at a concentration of 50 µM, resulted in a significant decrease in parasite load compared to the control group. However, only **3m** at 50 µM exhibited an inhibitory effect on the monolayer infection comparable to Bz at 100 µM (Figure 2). The results demonstrated a trypanostatic effect exhibited by **3g** and **3j**. These derivatives appear to modulate the release of trypomastigotes, likely by interfering with the parasite’s multiplication and differentiation processes. However, they were ineffective in eradicating the intracellular parasites present in the cell monolayer. In contrast, **3m** and Bz significantly inhibited the release of trypomastigotes and maintained a reduction in intracellular parasite load for up to 21 days post-infection (dpi). Despite these effects, neither compound achieved an in vitro sterile cure, which may be attributed to the need for prolonged treatment regimens or the persistence of dormant amastigotes.

### 2.2. Drug Toxicity and Efficacy in 3D Cardiac Spheroid

**Figure 3:**
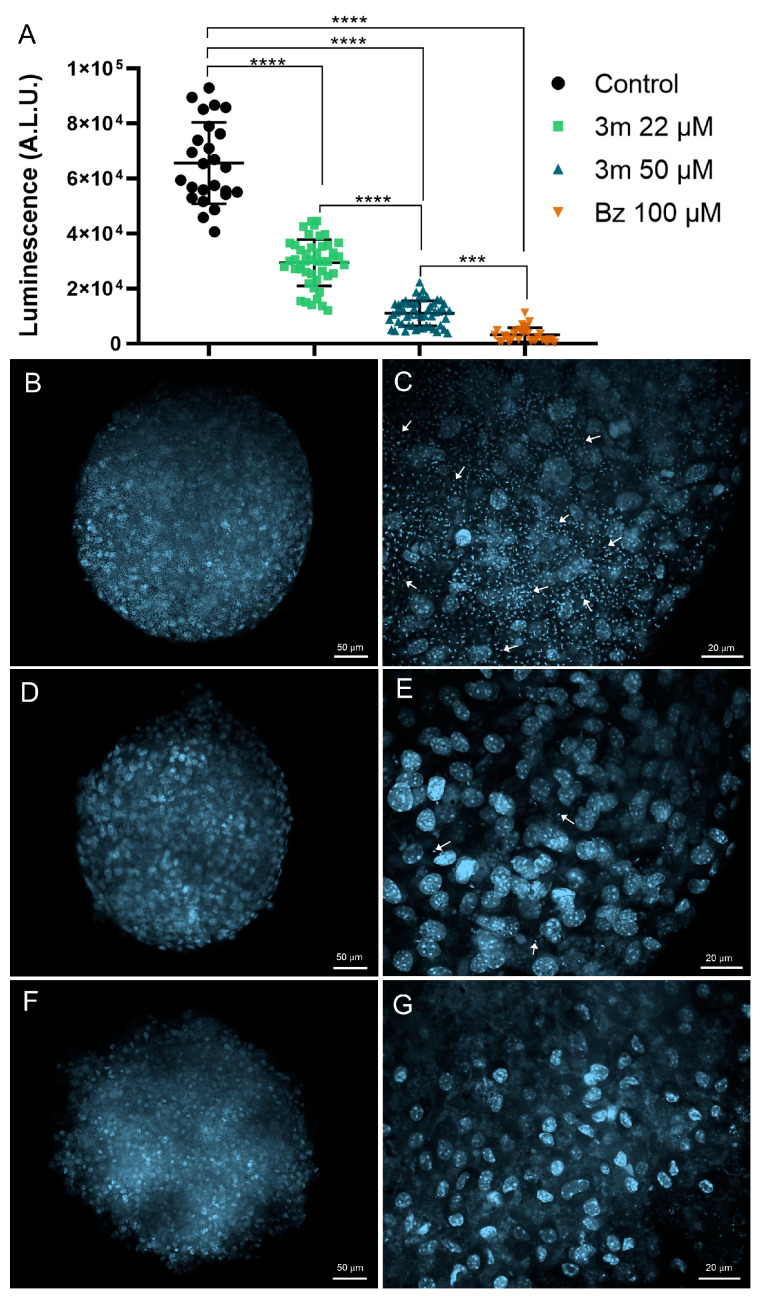
Efficacy of **3m** and Bz in *T. cruzi*-infected 3D cardiac spheroids. (**A**) Treatment with **3m** and Bz over 72 h resulted in a significant reduction in the parasite load, as measured by parasite viability expressed as Arbitrary Luminescence Units (A.L.U.) following the addition of luciferin (300 μg/mL). DAPI staining was performed on *T. cruzi*-infected 3D cardiac spheroids at 72 h (**B**,**C**) and after treatment with **3m** at 50 µM (**D**,**E**) or Bz at 100 µM (**F**,**G**). The host cell nuclei, as well as the nuclei and kinetoplast of the parasite (arrows), were visualized using the DNA dye. Data are presented as mean ± standard deviation from three independent experiments, each with at least six replicates. Statistical significance was assessed using One-way ANOVA followed by Tukey’s post hoc test, with significance levels indicated at *p* ≤ 0.001 (***) and *p* ≤ 0.0001 (****).

Following our selection of the **3m** as a promising candidate for advancement in the screening cascade, we assessed its toxicity and efficacy using three-dimensional (3D) primary cardiac spheroid models. This model closely mimics the organization and microenvironment of cardiac tissue, making it a valuable tool for evaluating drug penetration into microtissue and its efficacy [25]. A three-day treatment with **3m** exhibited low toxicity (Table 1) and significantly reduced the parasite load of 3D cardiac spheroids (Figure 3). The treatment of 3D cardiac spheroids with **3m** yielded a 50% cytotoxic concentration (CC_50_) value of 229.59 ± 2.93 µM, indicating a low cardiotoxic profile (Table 1). Bz treatment also demonstrated low toxicity in 3D cardiac spheroids, with a CC_50_ value exceeding 500 µM.

Given that cardiotoxicity is a major concern leading to the withdrawal of pharmaceutical agents from the market [26], these findings suggest a favorable safety profile, with no significant toxic effects detected in 3D cardiac cells. The compound **3m** significantly reduced the parasite load at concentrations of 22 µM and 50 µM (Figure 3). However, the 50 µM concentration of **3m** exhibited the highest efficacy in controlling infection, achieving a remarkable 81% inhibition (Figure 3). This inhibition level is comparable to Bz at 100 µM, resulting in a 95% inhibition rate. The substantial difference between the CC_50_ (229.59 µM) and **3m** at 50 µM, which achieves 80% efficacy (IC_80_), further supports its margin of safety.

Confocal microscopy images highlight the high reduction in the infection profile of 3D cardiac spheroids (Figure 3). The untreated 3D cardiac spheroids exhibit a high infection level, characterized by numerous intracellular parasites, which can be easily identified by staining the nucleus and kinetoplast with DAPI, a DNA dye. The exposure of 3D cardiac spheroids to 50 µM of **3m** resulted in a substantial decrease in the intracellular parasite load (Figure 3). The visualization of intracellular parasites in 3D spheroids treated with Bz is markedly reduced, with rare parasites observed (Figure 3).

The marked differences in cardiac overall architecture, physiology, and pharmacological responses between conventional two-dimensional (2D) cultures and three-dimensional (3D) environments have motivated the employment of 3D culture systems for elucidating biological processes and advancing drug development [27]. In particular, the cardiac spheroid model employed in this study effectively replicates in vivo conditions associated with Chagas disease, exhibiting key features such as cardiac hypertrophy and fibrosis [28]. Thus, this model has emerged as a powerful platform for elucidating pathological mechanisms and evaluating drug efficacy during the drug development process. In this context, our findings highlight the ability of **3m** to permeate the cardiac spheroid and demonstrate its potency in eliminating intracellular parasites.

### 2.3. Influence of 3m Absorption on Drug Efficacy

**Figure 4:**
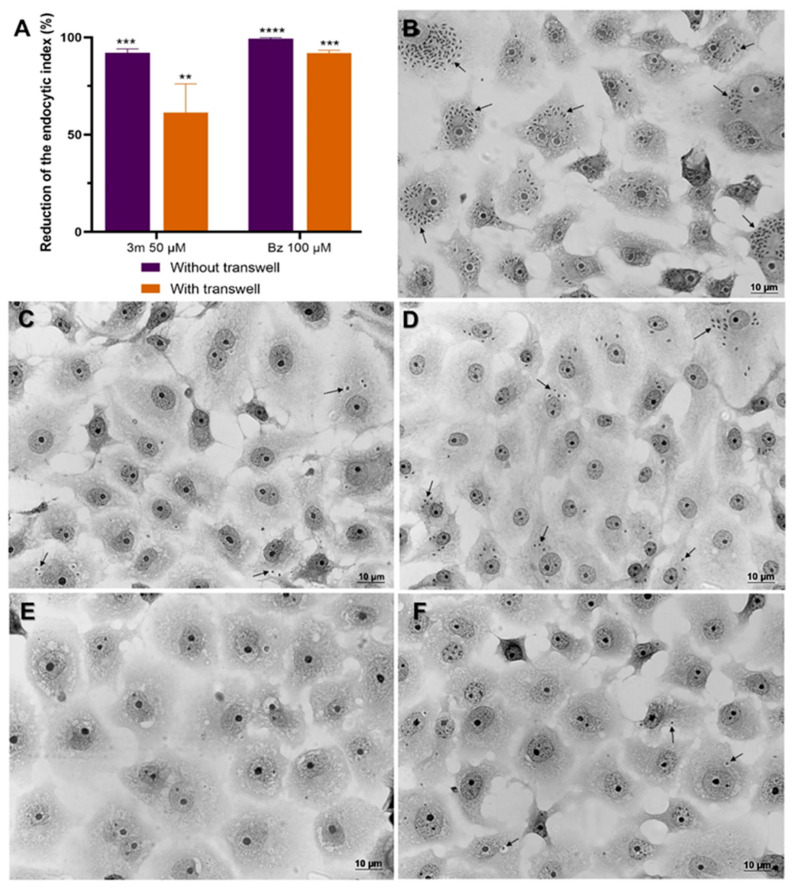
The analysis of drug absorption and efficacy using a Transwell system for co-culturing with Caco-2 cells and *T. cruzi*-infected Vero cells. (**A**) Treatment with **3m** and Bz significantly reduced the endocytic index in *T. cruzi*-infected Vero cells (Dm28c-Luc) when treatments were administered either on Caco-2 cell monolayers in the Transwell insert (indicated as with Transwell) or directly on Vero cell monolayers (termed without Transwell). Optical microscopy images of Vero cells infected with *T. cruzi* Dm28c-Luc after 72 h (**B**) and the subsequent effects following treatment with Bz (**C**) and **3m** (**D**) within the co-culture system (with Transwell). The impact of Bz (**E**) and **3m** (**F**) on *T. cruzi*-infected Vero cell cultures in the absence of the Transwell system is also presented. Black arrows indicate intracellular parasites. Statistical analysis was performed using Two-way ANOVA; *p*-values: *p* ≤ 0.01 (**), *p* ≤ 0.001 (***), and *p* ≤ 0.0001 (****). Bar = 10 μm.

To evaluate the impact of **3m** absorption on therapeutic efficacy in vitro, we performed a co-culture assay using Caco-2 cells and *T. cruzi*-infected Vero cells with a Transwell system. The confluent monolayers of Caco-2 cells, established on the Transwell membrane, were transferred to wells containing *T. cruzi*-infected Vero cells that had been infected for 24 h. The Caco-2 cells were then incubated with 50 μM of **3m** for 72 h, and the infection level in Vero cells was assessed to evaluate the effect of the compound. This co-culture system was evaluated in conjunction with a direct application of **3m** to infected Vero cells, bypassing the Transwell system. The efficacy of these treatments was assessed using the endocytic index, enabling a comparative analysis of their inhibitory effects. Our findings indicated that **3m** exhibited favorable permeability across the membranes of Caco-2 cells, leading to a decrease in the infection levels of Vero cell monolayers (Figure 4). Using a Transwell culture system with Caco-2 cells, we observed that **3m** treatment resulted in a significant 61% reduction in the endocytic index. Furthermore, when applied directly to infected Vero cells, the reduction effect was even more pronounced, reaching up to 91% (Figure 4). Treatment with Bz yielded a significant inhibitory impact, achieving a 99% reduction in the absence of the Transwell system and a 91% reduction with its implementation (Figure 4).

The high failure rate observed in in vivo preclinical trials for promising candidates in vitro emphasizes the need for more predictive cellular models in the early stages of drug development [23]. Absorption, a critical determinant of drug plasma concentration, plays a key role in pharmacokinetics [29]. Therefore, in vitro models are recommended for assessing the intestinal absorption and therapeutic efficacy of novel chemical entities. Caco-2 cells are widely regarded as the gold standard for mimicking the structural and functional characteristics of the human intestinal barrier, making them an essential platform for screening compounds that rely on passive transport mechanisms [30]. Our findings emphasize that **3m** effectively overcomes absorption barriers while maintaining significant efficacy in reducing parasite load under the evaluated experimental conditions.

### 2.4. Impact of the Combination on Drug Interaction

**Figure 5:**
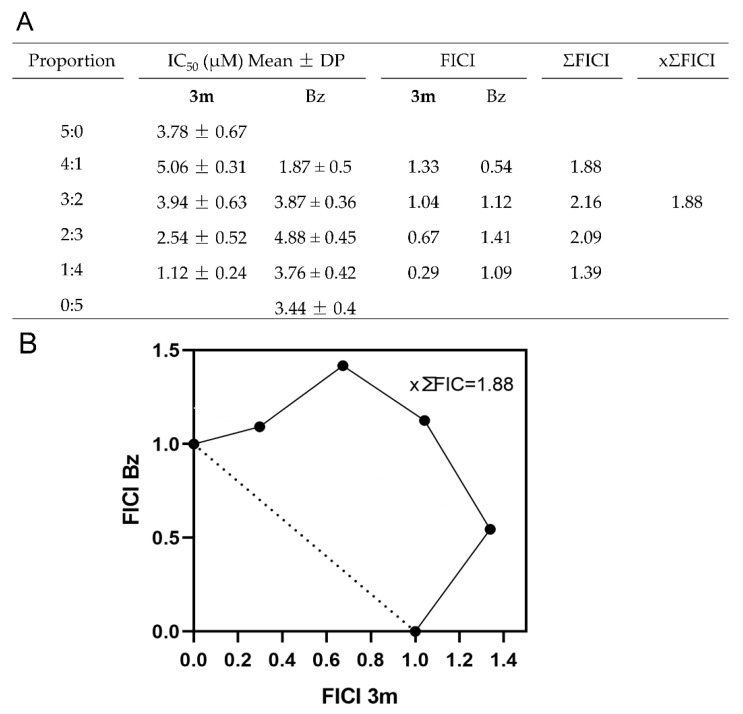
In vitro combination effect of **3m** and Bz. (**A**) The table shows the values for the fractional inhibitory concentration index (FICI), the sum of the FICI (ΣFICI), and the mean of the sum (xΣFICI). (**B**) The isobologram plot was constructed by analyzing the Fractional Inhibitory Concentration Index (FICI) values for varying ratios of **3m** in combination with Bz. xΣFIC <0.5 indicates synergistic effects, xΣFIC ranging from 0.5 to 4.0 indicates an additive effect, while xΣFIC >4.0 reflects an antagonistic effect. The isobologram was performed using FICI values, represented by a straight line. The dashed line represents the theoretical values of the additive effect of the combinations.

The strategic combination of drug candidates with Bz is designed to enhance therapeutic efficacy and safety, addressing the inherent limitations of monotherapy in chronic Chagas disease [31]. This combinatorial approach targets multiple pathways within the parasite, representing a promising strategy to counteract resistance mechanisms observed in certain *T. cruzi* strains [32]. Thus, we conducted an in vitro assessment of the interaction between Bz and **3m** using the fixed-ratio isobologram method [33]. Based on the IC_50_; values, the maximum concentrations of the compounds were determined: 125 µM for derivative **3m** and 95 µM for Bz. From these concentrations, the following combination ratios were defined: 5:0 (125 µM), 4:1 (100:19 µM), 3:2 (75:38 µM), 2:3 (50:57 µM), 1:4 (25:76 µM), and 0:5 (0:95 µM). In Vero cell cultures infected with *T. cruzi* (Dm28c-Luc), the combined treatment with Bz and **3m** showed summed fractional inhibitory concentrations (ΣFIC) values between 1.39 and 2.16 (Figure 5A). According to these results, all tested ratios fall into the additive effect category (ΣFIC > 0.5 and 4.0). Analysis of the isobologram, together with the mean ΣFIC value (xΣFIC = 1.88; Figure 5A,B), reinforces the additive nature of the interaction for this combination of compounds, demonstrating that the observed efficacy corresponds to the cumulative impact of each compound [34].

Implementing drug combination regimens is an important strategy for overcoming the limitations of existing therapies for many neglected tropical diseases [35]. Such strategies are designed to minimize the dosage of each agent, potentially leading to improved tolerability during treatment [36]. When assessing the in vitro additive interactions between the sulfone metabolite of fexinidazole (fex-SFN) and Bz in the context of a *T. cruzi* in vivo infection model, remarkable enhancements in cure rates and a rapid decline in parasitemia were observed, compared to monotherapy, with no discernible toxicity [37]. Furthermore, the in vitro combination of NDP-227, a pyrazolone derivative, with Bz demonstrated significant improvements in the murine model of acute *T. cruzi* infection, resulting in a reduction of more than 87% in parasitemia and an elevated survival rate of greater than 83%. These outcomes surpassed those achieved with Bz or NDP-227 used as monotherapy treatments [38]. These promising results warrant further investigation into the potential effect of combining Bz with **3m**, particularly in vivo models of acute infection, compared to monotherapy.

### 2.5. T. cruzi Acute Experimental Infection

**Figure 6:**
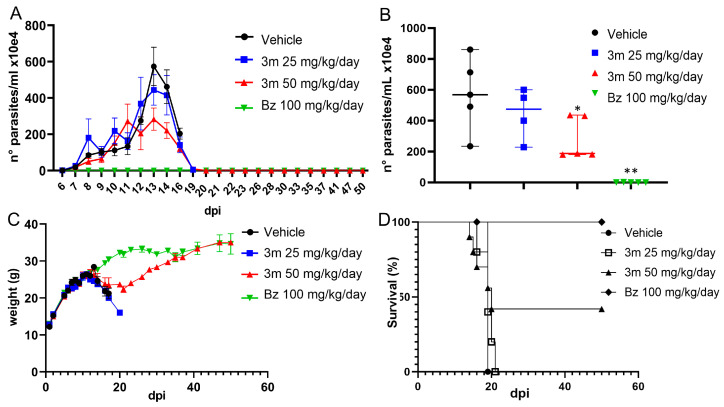
Impact of **3m** treatment on mice acutely infected with *T. cruzi*. Parasitemia (**A**), weight (**C**) curves, and survival rates (**D**) in infected untreated, **3m**-treated (25 and 50 mg/kg/day), and Bz-treated (100 mg/kg/day) mice were monitored up to 50 days post-infection (dpi). A significant 50% reduction in parasite load was noticed on the parasitemia peak (**B**). Statistical significance was assessed relative to the control group using the Mann–Whitney test. *p* ≤ 0.05 (*), and *p* ≤ 0.01 (**).

In this study, Swiss Webster male mice were used to assess the efficacy of **3m** in *T. cruzi* acute infection (Y strain). We first determined the no-observed-adverse-effect level (NOAEL) of **3m** in male mice, conducting a series of consecutive oral administrations at a dosage of 50 mg/kg every hour. Side effects were only observed at cumulative doses exceeding 350 mg/kg. Based on the NOAEL findings, we selected dosages of 25 and 50 mg/kg/day for post-infection treatment, using 1% solutol solution as the vehicle. Mice that exhibited positive parasitemia by day 5 post-infection received oral treatments of either 25 mg/kg/day or 50 mg/kg/day of **3m**, or 100 mg/kg/day of Bz, administered in two divided doses over a five-day duration. Daily evaluations included monitoring blood parasitemia levels, weight changes, and signs of distress. Our findings indicated a slight reduction in parasitemia in the group receiving 25 mg/kg/day of **3m**, with a more significant suppression of the parasites observed at the higher dose of 50 mg/kg/day, resulting in a 50% reduction in the peak of parasitemia (Figure 6). In contrast, Bz treatment decreased the overall parasite load more effectively. Circulating parasites were monitored up to 50 dpi, even when parasitemia had declined (16–19 dpi). Weight assessments showed a decline in the vehicle-untreated and low-dose **3m** groups. However, Bz-treated mice prevented weight loss. By the end of the treatment, mice treated with 50 mg/kg/day of **3m** approached weights like those in the Bz group (Figure 6). Survival rates highlighted differences between treatment groups, with vehicle-treated and low-dose **3m** groups experiencing high mortality rates, reaching 100% mortality by 19 and 21 dpi, respectively (Figure 6). In contrast, a 40% survival rate was noted in mice treated with a higher dose of **3m**, while all mice in the Bz treatment group survived (Figure 6).

**Figure 7:**
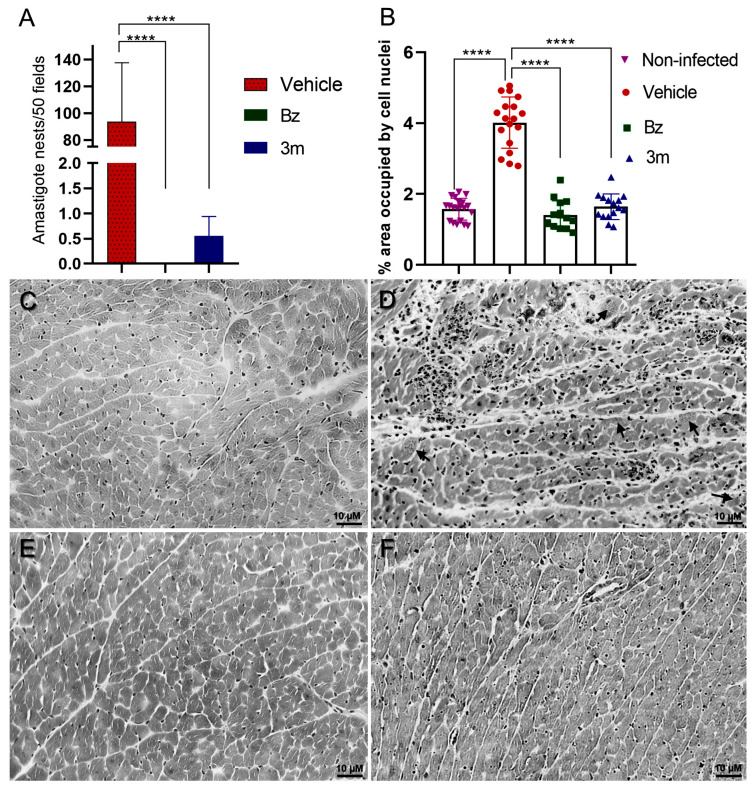
Histopathological analysis of mouse myocardium was performed, focusing on H&E staining of cardiac tissue from acute *T. cruzi* infection models. (**A**) Amastigote nest quantification was performed across 50 microscopic fields. (**B**) The extent of tissue area occupied by inflammatory infiltrates was analyzed using the ImageJ software (version 1.54p). (**C**) Uninfected control samples served as baseline measurements for comparison. Cardiac tissue from untreated (**D**) and *T. cruzi*-infected mice treated with **3m** at 50 mg/kg/day (**E**) and Bz at 100 mg/kg/day (**F**). Results are presented as the mean ± standard deviation. Statistical significance was assessed using One-way ANOVA followed by Tukey’s post hoc test, with significance levels indicated at *p* ≤ 0.0001 (****). Bar = 10 µm.

These findings have driven an investigation into the potential for favorable prognoses regarding cardiac tissue damage, particularly tissue parasitism and the inflammatory infiltrate in the surviving animals. Consequently, we carried out a histological analysis of myocardial tissue samples from surviving mice treated with Bz and **3m**. These results were compared with samples from an untreated control group during the phase of parasitemia decline. The quantitative analysis demonstrated a significant reduction in amastigote nests and inflammatory infiltrates in cardiac tissue treated with Bz and **3m** (Figure 7). The *T. cruzi*-infected mice exhibited a markedly high tissue infection level and pronounced myocarditis. An average of 93.66 ± 44 amastigote nests per 50 microscopic fields was found in the cardiac tissue of untreated animals (Figure 7). In contrast, cardiac tissue samples treated with **3m** exhibited significantly reduced infection level, with a mean of only 0.55 ± 0.3 parasite nests, indicating control of the tissue parasitism. Furthermore, no parasites were visualized on Bz-treated animal tissues (Figure 7).

This phenomenon was visualized in cryosections of cardiac tissue from both untreated mice and those exposed to **3m** or Bz treatment (Figure 7). Furthermore, the groups of treated animals (**3m** and Bz) also displayed a distinct profile of inflammatory infiltrate in the cardiac tissue compared to the control group. As expected, intense inflammatory infiltrates were observed in *T. cruzi*-infected and untreated mice, with a 2-fold increase in inflammatory cells per tissue area. Both Bz and **3m** treatments significantly reduced the level of inflammatory infiltrates, which returned to baseline levels seen in uninfected mice (Figure 7).

**Figure 8:**
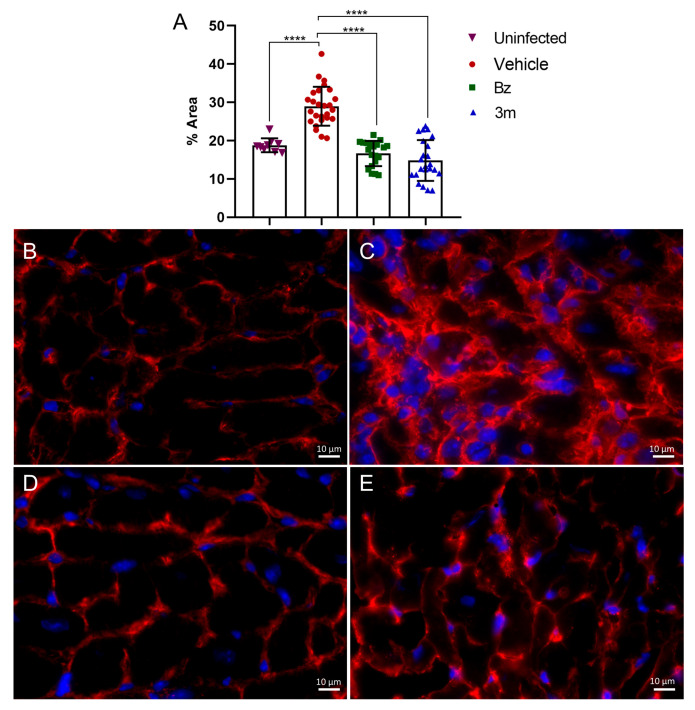
Fibronectin immunofluorescence (shown in red) was analyzed in cardiac tissues from Swiss Webster mice, both infected and uninfected with *T. cruzi*. The percentage of fibronectin expression area is presented in panel (**A**). Representative images at 63x magnification are shown for the uninfected cardiac tissue (**B**) as well as for untreated (**C**) and treated *T. cruzi*-infected mice receiving **3m** at 50 mg/kg/day (**D**) and Bz at 100 mg/kg/day (**E**). The cell nuclei were stained with DAPI (shown in blue). Statistical significance was assessed relative to the control group using One-way ANOVA followed by Tukey’s post hoc test, with *p*-values ≤ 0.0001 (****). Bar = 10 µm.

Additionally, our findings demonstrated a positive impact on cardiac fibrosis. In the myocardium of infected and untreated mice, fibronectin labeling showed marked deposition, characterized by prominent thick fibronectin fibers within the cardiac interstitium (Figure 8). In contrast, uninfected control animals exhibited thin interstitial fibronectin fibers at the cell–cell interface. The extracellular matrix (ECM) changes in infected mice indicated a significant 1.6-fold increase in fibronectin levels (Figure 8). Administering **3m** at 50 mg/kg/day effectively prevented fibronectin accumulation in the cardiac interstitium, restoring the ECM profile to resemble that of uninfected mice and those treated with Bz. Treatment with **3m** resulted in a significant reduction in fibronectin deposition within the cardiac tissue, closely matching the fibronectin level in uninfected tissue and the effects seen with Bz treatment (Figure 8). Fluorescence images demonstrated the impact of **3m** and Bz treatment on fibronectin fiber deposition in cardiac tissues, showing a thin layer similar to that observed in the uninfected control group (Figure 8). Therefore, reducing the parasite burden with **3m** treatment leads to positive outcomes, attenuating the pathological effects.

Treatment with lead compounds from the 4-aminopyridyl series, targeting CYP51, has significantly suppressed parasitemia and reduced myocarditis in *T. cruzi*-infected mice [39]. Although no sterile cure was achieved, the treatment yielded improvements in survival rates, hepatoprotection, and reductions in inflammatory infiltrates during the acute phase of *T. cruzi* (Y strain) infection. In a chronically infected murine model, these compounds showed promise in mitigating myocardial damage. However, the suppression of parasitemia was not sustained following cycles of immunosuppression. Additionally, the combination therapy of Bz and amiodarone (AMD) has effectively mitigated the pathological manifestations of experimental acute Chagas disease, restoring cardiac function by reducing parasite load, inflammation, and fibrosis [40]. The Bz/AMD combination prevented alterations in cardiac electrical conduction and improved survival rates in mice, highlighting the potential of AMD as a valuable candidate for combined treatment with Bz. Furthermore, a multi-therapeutic approach combining a suboptimal dose of Bz with the immunomodulatory agent Pentoxifylline (PTX) also demonstrated a positive impact on the prognosis of chronic Chagas cardiomyopathy in Colombian-infected C57BL/6 mice, resulting in a significant reversal of chronic cardiac pathologies [41]. Thus, the combination of **3m** with suboptimal doses of Bz has the potential to achieve a parasitological cure while also improving the pathological features of the disease.

## 3. Materials and Methods

### 3.1. Chemistry

The compounds **3g**, **3j**, and **3m** were synthesized according to the experimental protocol previously published by our research group [22]. The chemical structures were confirmed by Fourier Transform Infrared (FT-IR) spectroscopy, Nuclear Magnetic Resonance (NMR), and High-Resolution Mass Spectrometry (HRMS). The analytical results are exhibited below.

1-(4-chlorophenyl)-4-(4,5-dihydro-1*H*-imidazol-2-yl)-1*H*-pyrazole (**3g**)

Yield: 78%; m.p.: 214–216°C; FT-IR ν (cm*^−^*^1^): 3146, 3109, 3075, 2935, 2879, 1630, 1601, 1566, 1504, 1486; ^1^H NMR (500 MHz, DMSO-*d6*) δ 8.83 (s, 1H), 8.04 (s, 1H), 7.88 (d, *J* = 8.9 Hz, 2H), 7.59 (d, *J* = 8.9 Hz, 2H), 3.56 (s, 4H); ^13^C NMR (125 MHz, DMSO-*d_6_*) δ 157.5, 140.3, 138.0, 130.8, 129.5, 127.5, 120.1, 116.3, 49.1; HRMS (ESI) *m/z* [M + H]^+^ = 247.0749 (found), [M + H]^+^ = 247.0750 (calculated).

1-(4-bromophenyl)-4-(4,5-dihydro-1*H*-imidazol-2-yl)-1*H*-pyrazole (**3j**)

Yield: 65%; m.p.: 228–230°C; FT-IR ν (cm*^−^*^1^): 3144, 3110, 3066, 2931, 2863, 1632, 1589, 1565, 1485; ^1^H NMR (500 MHz, DMSO-*d6*) δ 8.83 (s, 1H), 8.04 (s, 1H), 7.81 (d, *J* = 8.8 Hz, 2H), 7.72 (d, *J* = 8.8 Hz, 2H), 3.56 (s, 4H); ^13^C NMR (125 MHz, DMSO-*d6*) δ 157.5, 140.3, 138.4, 132.4, 127.5, 120.4, 119.0, 116.4, 49.1; HRMS (ESI) *m/z* [M + H]^+^ = 291.0256 (found), [M + H]^+^ = 291.0245 (calculated).

1-(3-chloro-4-methylphenyl)-4-(4,5-dihydro-1H-imidazol-2-yl)-1H-pyrazole (**3m**)

Yield: 92%; m.p.: 168–170°C; FT-IR ν (cm*^−^*^1^): 3199, 3122, 2941, 2879, 1627, 1604, 1583, 1564, 1509, 1488; ^1^H NMR (500 MHz, DMSO-*d6*) δ 8.85 (s, 1H), 8.02 (s, 1H), 7.92 (d, *J* = 2.2 Hz, 1H), 7.74 (dd, *J* = 8.3, 2.2 Hz, 1H), 7.50 (d, *J* = 8.3 Hz, 1H), 3.55 (s, 4H), 2.36 (s, 3H); ^13^C NMR (125 MHz, DMSO-*d6*) δ 157.5, 140.2, 138.2, 134.0, 133.7, 132.1, 127.6, 118.6, 117.0, 116.2, 49.2, 19.0; HRMS (ESI) *m/z* [M + H]^+^ = 261.0911 (found), [M + H]^+^ = 261.0907 (calculated).

### 3.2. Parasites

The experimental protocols employed genetically modified *T. cruzi* expressing the luciferase gene (Dm28c-Luc), generously provided by Dr. Cristina Henriques [42]. The parasites were cultured in Vero cells using RPMI-1640 medium (Sigma-Aldrich, St. Louis, MO, USA) supplemented with 10% fetal bovine serum (FBS) (Cultilab, Campinas, SP, Brazil) at 37°C in a humidified atmosphere with 5% CO_2_. Four days post-infection (4 dpi), the parasites were harvested from the culture supernatant for subsequent in vitro assays. For in vivo studies, the *T. cruzi* Y strain was isolated through cardiac puncture from *Swiss Webster* mice at peak parasitemia (7 dpi), as previously described [43].

### 3.3. Cell Culture

Vero cells, acquired from the Cell Bank of Rio de Janeiro, were cultured in RPMI medium supplemented with 10% fetal bovine serum (FBS). Cultures were maintained at 37°C in a 5% CO_2_ atmosphere for experimental assays and parasite propagation.

Primary heart muscle cells were isolated from *Swiss Webster* mouse fetuses, as previously described [43]. The ventricle fragments underwent dissociation using trypsin and collagenase type II. The isolated cells were seeded at a density of 2.5 × 10^4^ cells per well in U-bottom plates pre-coated with 1% agarose [28]. These cells were cultured in Dulbecco’s Modified Eagle Medium (DMEM) (Sigma-Aldrich, St. Louis, MO, USA), supplemented with 7% FBS, 2.5 mM CaCl_2_, 2% embryo extract, 1 mM *L*-glutamine, and antibiotics. The cultures were maintained at 37°C in a humidified incubator with 5% CO_2_. All animal handling procedures were conducted per the ethical guidelines approved by the Animal Care and Use Committee of the Instituto Oswaldo Cruz (License L-017-2022-A2).

### 3.4. Cytotoxicity in Cardiac Muscle Cells

Three-dimensional cardiac spheroids, cultured for 7 days, were used to assess toxicological effects. The treatment with promising candidates was performed using serial dilutions ranging from 1.95 to 500 μM. After a 72-h incubation period at 37°C in a 5% CO_2_ atmosphere, 20 μL of CellTiter-Glo (Promega Corporation, Madison, WI, USA), which measures ATP levels, was added to each well. Luminescent signals were detected using a Glomax microplate reader (Promega Corporation, Madison, WI, USA). All experiments were conducted in duplicate and repeated three times to ensure reliability. The CC_50_ value, representing the concentration at which cell viability is reduced by 50%, was determined using GraphPad Prism software (version 8.2.1).

### 3.5. Evaluating the Drug’s Effectiveness on T. cruzi-Infected 3D Cardiac Spheroids

Three-dimensional cardiac microtissues were infected with 5 × 10^5^ trypomastigotes of *T. cruzi* Dm28c-Luc per spheroid. After 24 h, the spheroids were washed with their culture medium to remove non-internalized parasites. The treatment was performed at IC_90_ and twice the IC_90_ concentrations of the promising candidates. The positive control was Bz (100 μM), with DMSO (≤ 1%) as the negative control. After 72 h of incubation, a *D*-Luciferin solution (300 μg/mL) was added, and the luminescence signal was measured using a Glomax microplate reader. After reading out, the spheroids were fixed for 20 min at 4°C in 4% paraformaldehyde in PBS. The intracellular parasites were assessed using 4′,6-diamidino-2-phenylindole (DAPI), a DNA dye, and images were acquired using a Zeiss confocal microscope LSM 710 (Carl Zeiss, Baden–Württemberg, Germany) [44].

### 3.6. Washout Assay

Vero cell monolayers were infected with *T. cruzi* Dm28c-Luc at a parasite-to-host cell ratio of 10:1 for 24 h. After washing, the promising candidate was added at concentrations corresponding to IC_90_ and 50 µM, then incubated for 10 days. Following this 10-day treatment, the monolayers were washed three times with PBS to ensure complete removal of the compound. The culture was then maintained for an additional 10 days without treatment. Every 3 to 4 days, the culture supernatant was collected and analyzed for viable parasites by adding luciferin; the medium without the compound was replaced afterward. After 21 days, the monolayer was evaluated for parasite load using the Glomax microplate reader (Promega Corporation, Madison, WI, USA). Bz (100 μM) served as a positive control, and DMSO (≤1%) as a negative control [44].

### 3.7. Drug Absorption and Efficacy

A co-culture model, using Caco-2 cells grown on Transwell inserts and *T. cruzi*-infected Vero monolayers grown on coverslips, was employed to assess the absorption and efficacy of the promising candidate. Caco-2 cells were seeded at a density of 8 × 10^4^ cells per insert and allowed to grow for 7 days until reaching confluence. After infecting the Vero cell culture for 24 h, the Caco-2 cell cultures (inserts) were transferred to the Vero cell culture plate. The treatment was applied in two ways: first, by introducing effective and non-cytotoxic concentrations of the promising candidate to Caco-2 cells in Transwell inserts; second, by directly exposing the Vero cell monolayers infected with *T. cruzi*. After 72 h of treatment, the Vero cell monolayers on the coverslips were fixed in Bouin’s solution for 5 min at room temperature, then subjected to Giemsa staining. The coverslips were washed with distilled water, dehydrated using acetone/xylene solutions, and mounted with Permount. The endocytic index (EI) was calculated by multiplying the average percentage of infected cells by the average number of parasites per cell, determined after counting at least 200 cells/coverslip. The experiment was conducted in duplicate and repeated three times. Images were acquired under a Zeiss Axio Imager M2 microscope using the Axio Vision software (version 4.8).

### 3.8. Drug Combination Assay

The combined impact of the promising candidate and Bz was assessed using the isobologram method [33]. Vero cells infected with *T. cruzi* Dm28c-Luc were exposed to a 72-h treatment at 37°C with varying ratios of Bz/ promising candidate (5:0, 4:1, 3:2, 2:3, 1:4, 0:5). The maximum concentrations of the compounds were determined to ensure their 50% inhibitory concentration (IC_50_) values fell within the midpoint of the dilution range. The IC_50_ value for each ratio and compound was calculated individually. The fractional inhibitory concentration index (FICI) was calculated as the ratio of the compound’s IC_50_ in the combination to its IC_50_ alone. The sum of the FICI (ΣFICI) was determined as the composite FICI A + composite FICI B. The average of the sums of FICI, xΣFICI, was used to classify the interaction as synergistic (xΣFICI ≤ 0.5), additive (xΣFICI > 0.5–4), and antagonistic (xΣFICI > 4) [34]. The isobologram graph was constructed by plotting the FICI.

### 3.9. In Vivo Assay

Swiss Webster male mice, six weeks old, were obtained from the Institute of Science and Technology in Biomodels (FIOCRUZ, Rio de Janeiro, RJ, Brazil). The mice were housed in groups of up to four animals per cage within a standard room kept at a temperature range of 20 to 24°C, following a 12 h light and 12 h dark cycle. Cage enrichments were rotated weekly to guarantee animal welfare. Unrestricted access to standard chow and water was provided to the mice. All animal-related procedures were conducted following the guidelines of the IOC/Fiocruz Institutional Animal Care and Use Committee, License L-017-2022-A2 to Mirian Claudia de Souza Pereira.

Before infection assays, we performed the no-observed-adverse-effect level (NOAEL) test for the compound, following the Guidelines for testing chemicals from the OECD [45]. Swiss Webster male mice received the promising candidate at a dose of 50 mg/kg, administered at 100 μL/hour, formulated in 1% solutol (also known as Kolliphor HS15) (Sigma-Aldrich, St. Louis, MO, USA).

In the experimental acute infection model, male *Swiss Webster* mice were intraperitoneally infected with 10^4^ bloodstream trypomastigotes of *T. cruzi Y* strain. After 5 days of infection, parasitemia was evaluated using the Pizzi-Brenner method [46], and mice with negative parasitemia were excluded from the study. The mice with confirmed positive parasitemia were randomly assigned to the experimental groups (*n* = 5 animals per group divided into two cages): (1) Infected/Untreated (Vehicle-Solutol), (2 and 3) two non-toxic doses (mg/kg/day) determined by the preceding NOAEL test, and (4) Bz 100 mg/kg/day. Treatment was administered via gavage b.i.d at 12 h intervals for 5 days. To minimize observation bias (blinding), all daily measurements (parasitemia monitoring, body weight tracking, and survival assessment) were performed by a technician who was unaware of the animal groupings and corresponding treatments. Parasitemia was monitored daily for 30 days, then every three days, while body weight and survival rates were tracked throughout the entire 50-day study period. Uninfected and treated animals were used as toxicity controls for compounds.

### 3.10. Histological Analysis

At the end of the in vivo assay, at 50 dpi, the animals were humanely euthanized, and their hearts were collected and cryopreserved using Tissue-Tek O.C.T. solution for subsequent histological sectioning in a LEICA cryostat. The histological sections were stained with Hematoxylin and Eosin (H&E) following the Harris method [47]. Amastigote nest quantification was performed by counting nests across 50 microscopic fields, with three animals per group. The assessment of inflammatory infiltrates was performed using Fiji software (version 2.9.0), which involved segmenting cell nuclei and calculating the percentage of the total image area occupied by these nuclei [39]. For this analysis, at least five images per animal (three animals per group) were captured at 10x magnification.

After cryosectioning, the samples also underwent indirect immunofluorescence processing. They were initially rinsed in PBS and then incubated for 20 min in PBS with 4% bovine serum albumin (BSA). After incubating the tissue overnight at 4°C with either anti-fibronectin or anti-collagen I antibodies (1:200) (Sigma), the samples were washed and then subjected to incubation with TRITC-conjugated anti-rabbit IgG antibody (Sigma) (1:400). After washing, the samples were stained with 4′,6-diamidino-2-phenylindole (DAPI) and mounted with 2.5% 1,4-diazabicyclo [2.2.2]octane (DABCO). Images were captured using a Zeiss Axio Imager M2 fluorescence microscope with Axio Vision software (version 4.8).

### 3.11. Statistical Analysis

The data were summarized as mean ± standard deviation from three independent experiments. Statistical analyses were performed using the GraphPad Prism software (version 8.2.1.). For comparisons involving three or more groups with normal distribution and equal variances (washout assays, anti-*T. cruzi* efficacy in the 3D model, histological quantification analyses, and absorption and efficacy assays), a One-way ANOVA followed by Tukey’s post hoc test was used to determine significant differences. The Two-way ANOVA was employed for the absorption and efficacy assays in the Transwell system. For comparisons of two groups in the in vivo model (parasitemia), the Mann–Whitney U test was employed. A *p*-value ≤ 0.05 was considered statistically significant.

## 4. Conclusions

The results of this study underscore the therapeutic promise of the pyrazole-imidazoline derivative **3m** as a candidate for Chagas disease treatment. This compound exhibited low cytotoxicity, favorable permeability in an intestinal barrier model, and consistent antiparasitic effectiveness both in vitro and in vivo. Remarkably, treatment with **3m** led to significant reductions in parasite burden, inflammatory infiltrate, and cardiac fibrosis, while also improving survival rates in the acutely infected mouse model. Although a sterile cure was not confirmed, the findings suggest that monotherapy may be insufficient for complete parasite eradication. Therefore, exploring combination therapy involving **3m** and sub-therapeutic doses of Bz may represent a viable strategy to augment the antiparasitic effectiveness and overcome the limitations of currently available treatments. Further investigations are necessary to advance preclinical in vivo evaluations and refine therapeutic strategies against Chagas disease.

## Figures and Tables

**Table 1:** Toxic effect of pyrazole-imidazoline derivative (**3m**) on 3D cardiac spheroid.

**Compounds**	**Toxicity (CC_50_ µM)**	
	**3m**	**Bz**
	229.59 ± 2.93	>500

CC_50_ means values from three independent experiments ± standard deviation (SD); CC_50_: concentration that reduces the viability of cells by 50%.

## Data Availability

The original contributions presented in this study are included in the article. Further inquiries can be directed to the corresponding author.

## Abbreviations

The following abbreviations are used in this manuscript:

AMD	Amiodarone
BSA	Bovine Serum Albumin
Bz	Benznidazole
CD	Chagas disease
DABCO	1,4-diazabicyclo[2.2.2]octane
DAPI	4′,6-diamidino-2-phenylindole
DMEM	Dulbecco’s Modified Eagle Medium
dpi	Days post-infection
ECM	Extracellular Matrix
DMSO	Dimethyl sulfoxide
EI	Endocytic Index
FBS	Fetal Bovine Serum
FICI	Fractional Inhibitory Concentration Index
FT-IR	Fourier Transform Infrared
H&E	Hematoxylin & Eosin
HRMS	High-Resolution Mass Spectrometry
Nif	Nifurtimox
NOAEL	No-oberserved-adverse-effect level
NMR	Nuclear Magnetic Resonance
PDB	Protein Data Bank
pIC50	Negative log of IC50 in molar
PTX	Pentoxifyline
SI	Selectivity index
WHO	World Health Organization
